# Strengthening Laboratory Management Towards Accreditation: The Lesotho experience

**DOI:** 10.4102/ajlm.v1i1.9

**Published:** 2012-05-30

**Authors:** David Mothabeng, Talkmore Maruta, Mathabo Lebina, Kim Lewis, Joe Wanyoike, Yohannes Mengstu

**Affiliations:** 1 Ministry of Health and Social Welfare, Maseru, Lesotho; 2 Clinton Health Access Initiative, Maseru, Lesotho; 3 Association of Public Health Laboratories, Maseru, Lesotho; 4 Centers for Disease Control and Prevention, Maseru, Lesotho

## Abstract

**Introduction:**

The Lesotho Ministry of Health and Social Welfare’s (MOHSW) 5-year strategic plan, as well as their national laboratory policy and yearly operational plans, directly addresses issues of accreditation, indicating their commitment to fulfilling their mandate. As such, the MOHSW adopted the World Health Organization Regional Headquarters for Africa’s Stepwise Laboratory Quality Improvement Toward Accreditation (WHO–AFRO–SLIPTA) process and subsequently rolled out the Strengthening Laboratory Management Towards Accreditation (SLMTA) programme across the whole country, becoming the first African country to do so.

**Methods:**

SLMTA in Lesotho was implemented in two cohorts. Twelve and nineteen laboratory supervisors and quality officers were enrolled in Cohort 1 and Cohort 2, respectively. These 31 participants represented 18 of the 19 laboratories nationwide. For the purposes of this programme, the Queen Elizabeth II (QE II) Central Laboratory had its seven sections of haematology, blood bank, cytology, blood transfusion, microbiology, tuberculosis laboratory and chemistry assessed as separate sections. Performance was tracked using the WHO–AFRO-SLIPTA checklist, with assessments carried out at baseline and at the end of SLMTA. Two methods were used to implement SLMTA: the traditional ‘three workshops’ approach and twinning SLMTA with mentorship. The latter, with intensive follow-up visits, was concluded in 9 months and the former in 11 months. A standard data collection tool was used for site visits.

**Results:**

Of the 31 participants across both cohorts, 25 (81%) graduated (9 from Cohort 1 and 16 from Cohort 2). At baseline, all but one laboratory attained a rating of zero stars, with the exception attaining one star. At the final assessment, 7 of the 25 laboratories examined at baseline were still at a rating of zero stars, whilst 8 attained one star, 5 attained two stars and 4 attained three stars. None scored above three stars. The highest percentage improvement for any laboratory was 51%, whereas the least improved dropped by 6% when compared to its baseline assessment. The most improved areas were corrective actions (34%) and documents and records (32%). Process improvement demonstrated the least improvement (10%).

**Conclusion:**

The SLMTA programme had an immediate, measurable and positive impact on laboratories in Lesotho. This success was possible because of the leadership and ownership of the programme by the MOHSW, as well as the coordination of partner support.

## Introduction

The Lesotho Ministry of Health and Social Welfare (MOHSW), through its Laboratory Services Directorate, is committed to the provision of essential services as part of the national health-care delivery to all Basotho people. These services include comprehensive diagnostic testing of all prevalent major infections, for example, HIV and TB, monitoring of patient treatment, drug resistance testing and surveillance studies that inform policymaking decisions and major health reform.^1^

The laboratory system in Lesotho is structured in three tiers: referral, regional and district laboratories. The Central Laboratory at the Queen Elizabeth II (QE II) Hospital in Maseru serves as the national reference laboratory. There are two regional laboratories. The other 16 laboratories are at the district level, with 7 of these managed by the MOHSW, 1 by the military and 7 by the Christian Health Association of Lesotho. The final district laboratory is owned by the Partners in Health, anon-governmental organisation.

There has been a significant and progressive increase in demand for laboratory services in Lesotho, with the QE II Central Laboratory testing 114 114 specimens in 2006, compared to 16 250 in 2003, a 600% increase.^2^ The MOHSW realised that the increased demand for testing had to be matched with high quality testing. Accreditation was identified as one means of assuring continuous quality testing services.^3^

The MOHSW and its laboratory partners have put in place a number of key pillars for launching their bid to accredit the public health and clinical laboratories successfully. These include a national laboratory policy, a 5-year national strategic plan, the appointment of a laboratory director and a national Quality Assurance Unit (QAU) headed by a national quality manager who is supported by key national quality officers. The QAU is a unit created by Laboratory Services to manage the quality improvement initiatives for the entire laboratory network. In the strategic plan, Objective 2.3 directly addresses these initiatives, aiming ‘to strengthen quality assurance of Laboratory Services and have a mechanism for attaining international accreditation defined’.^3^ These clearly stated objectives have been translated into yearly operational plans for the last 3 years,^2^ culminating, in 2011, in the generation of a ground breaking yearly operational plan that aimed to have seven laboratories declared ready for the World Health Organization Regional Headquarters for Africa Stepwise Laboratory Quality Improvement Toward Accreditation (WHO–AFRO–SLIPTA) process by the 3rd Quarter of that year.^2^ This target was achieved but, unfortunately, the WHO–AFRO–SLIPTA office was not ready to accept applications by that time. The QAU was therefore established to ensure continued support for these quality improvement efforts.

The Strengthening Laboratory Management Towards Accreditation (SLMTA) programme was launched concurrently with the stepwise WHO–AFRO–SLIPTA process in Kigali, Rwanda in 2009.^4^ SLMTA, a task-based curriculum, assists countries in the training of laboratory managers to implement the quality management system requirements of the WHO–AFRO–SLIPTA process, with the aim of granting them eventual international accreditation.^5^

The MOHSW of Lesotho immediately embraced the SLMTA programme soon after engaging in the trainers’ workshopheld at the African Centre of Integrated Laboratory Training in Johannesburg, South Africa in November 2009. This programme was implemented at an opportune time in Lesotho, because the country had already embarked on laboratory improvements through a number of policies and critical documents such as the national laboratory policy and 5-year strategic plan. A number of critical officers, namely the laboratory director, quality manager, national safety officer, national training officer, as well as programmes such as mentorship, were also in place. The SLMTA programme in Lesotho was coordinated by the MOHSW with the SLMTA coordinator facilitating as one of the quality officers within the QAU. A number of laboratory partners, namely the Association of Public Health Laboratories (APHL), the Centers for Disease Control and Prevention (CDC) and the Clinton Health Access Initiative (CHAI), have provided technical and logistical support to the programme. The SLMTA programme has been part of the Laboratory Services ’ yearly operational plans for the past 2 years.^2^

This paper describes the experience of Lesotho in implementing the SLMTA programme. The purpose is to share this experience and the lessons learned from it with other countries that are implementing, or planning to implement, the SLMTA programme. The analysis of the programme in this paper will also inform present programme activities in Lesotho, as plans for the training of more cohorts across the country are already underway.

## Methods

The SLMTA programme in Lesotho was implemented in two cohorts. The 2 cohorts ran simultaneously from January 2010 to January 2011, with 4 hospitals participating in both groups. Cohort 1 comprised 12 participants selected from 4 district laboratories, 7 QE II Central Laboratory sections (chemistry, haematology, cytology, microbiology, blood bank, blood transfusion and the TB laboratory) and 1 quality officer from the QAU. All four participants from district laboratories were laboratory managers, whilst three of the seven from the QE II Central Laboratory were section supervisors and the other four were section level quality officers (Table 1). Cohort 1 enrolled only laboratories that were located within the vicinity of Maseru, the location of the training venue.

**TABLE 1:** Profile of Cohort 1 and Cohort 2 participants in the Strengthening Laboratory Management Towards Accreditation (SLMTA) programme in Lesotho.

Laboratories tier classification	Laboratory name	Affiliations	Job title of participants	
			Cohort 1	Cohort 2
Queen Elizabeth II Central Laboratory (national reference laboratory)	Tuberculosis	Government	Quality officer^†^	Quality officer^†^
	Chemistry		Quality officer^†^	Quality officer
	Haematology		Acting supervisor	Supervisor^†^
	Cytology		Quality officer	–
	Blood bank		Quality officer	–
	Blood transfusion		Supervisor^†^	–
	Microbiology		Supervisor	–
Regional Laboratory	Ntsekhe	–	Supervisor
	Motebang	–	Supervisor
District laboratory	Makoanyane	Military	Supervisor	–
	St Joseph’s	CHAL	Supervisor	Quality officer
	Scott		–	Quality officer
	St James		–	Supervisor
	Paray		–	Supervisor
	Maluti		–	Supervisor
	Mamohau		–	Supervisor
	Tebellong		–	Supervisor^*^
	Seboche		–	Supervisor
	Mafeteng	Government	Supervisor	–
	Butha Buthe		–	Supervisor
	Machabeng		–	Supervisor
	Mokhotlong		–	Supervisor
	Berea		–	Supervisor
	Partners in Health	NGO	–	Supervisor
Quality Assurance Unit	–	–	–	Quality officer

† Did not graduate.

CHAL, Christian Health Association of Lesotho; NGO, non-governmental organisation.

Cohort 1 did not follow the three workshop series recommended for SLMTA; instead, SLMTA was twinned with mentorship, which was already under way. Seven of the participants in this cohort came from the QE II Central Laboratory, where the mentor, who was also the SLMTA facilitator, was conducting the second round of mentorship at that time. The other four were based within an 80 km radius of the Central Laboratory, whilst the participant from the QAU was based within the MOHSW headquarters, the training venue.

**FIGURE 1:**
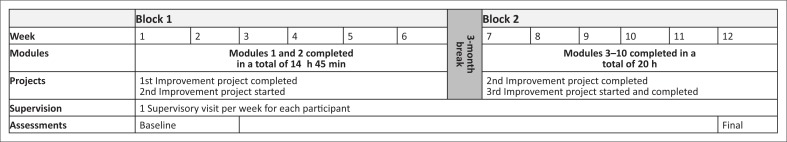
Schematic of the Strengthening Laboratory Management Towards Accreditation (SLMTA) Cohort 1 rollout.

Instead of the recommended 4–5 day workshops, the SLMTA modules were delivered 1 day per week on a Friday over two blocks of 6 weeks each. The two blocks were spaced 3 months apart. In total, the 1-day workshops over 12 weeks matched the 12 days of the recommended 4-day workshops, after the three-workshop series was completed (Figure 1). More intensive follow-ups were feasible because of the proximity of all participants. Each of the participants had one follow-up visit a week, to a total of 12 visits each over a 9-month period. The intensity of the follow-up visits allowed the participants to complete the recommended three improvement projects over the course of 9 months. The increased supervisory visits also allowed most of the participants to have more than one project running at a time; for example, if one was waiting for supply requisition documents from procurement, another project could be initiated on sample rejections.

**FIGURE 2:**
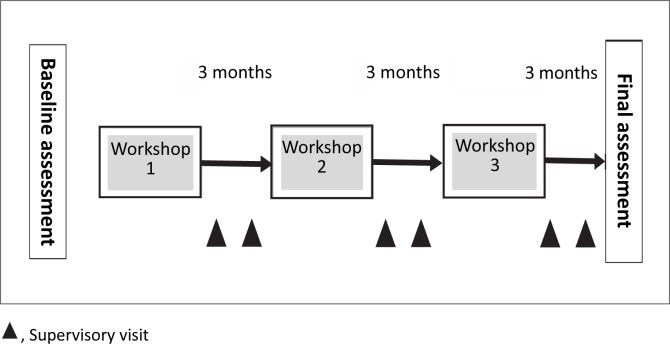
Strengthening Laboratory Management Towards Accreditation (SLMTA) Cohort 2 implementation model.

Cohort 2 followed the recommended SLMTA three workshops approach (Figure 2), with three certified SLMTA facilitators affiliated with the MOHSW, APHL and CHAI. A total of 16 district laboratories and 3 QE II Central Laboratory sections (each with 1 participant per laboratory, or per section, enrolled) were part of the SLMTA Cohort 2 (Table 1). Only one laboratory, Quithing District Laboratory, did not attend the training because the communication for workshop attendance did not arrive on time.

An implementation plan was developed before the programme started to ensure that the SLMTA programme for Cohort 2 had specific, fixed dates of activities spanning the entire 12 months. These were adhered to 98% of the time; that is, only one of the planned activities did not take place on the assigned date. The last activity not met was the assessment by WHO for the SLIPTA star status recognition, as the structures for such assessments were not in place by March 2010 (Table 2).

**TABLE 2:** Lesotho Strengthening Laboratory Management Towards Accreditation (SLMTA) Cohort 2 implementation plan, developed before the start of the programme.

Period	Activity
January 2010 – March 2010	Baseline assessments
April 2010	Workshop #1 + IPs
May 2010	Follow-up supportive visits + IPs
July 2010	Follow-up supportive visits + IPs
August 2010	Workshop #2 + IPs
September 2010	Follow-up supportive visits + IPs
October 2010	Follow-up supportive visits + IPs
November 2010	Workshop #3 + IPs Follow-up supportive visits + IPs
January 2011 – February 2011	Follow-up supportive visits + IPs Final assessments All participants meeting: final IP reports and final assessment report Assessment by external assessors for seven top performing laboratories
March 2011	Assessments by WHO–AFRO–SLIPTA assessors for top seven performing laboratories

IP, improvement project; WHO, World Health Organization; WHO–AFRO–SLIPTA, World Health Organization Regional Headquarters for Africa Stepwise Laboratory Quality Improvement Toward Accreditation.

### Baseline assessments

Baseline assessments were conducted by the three SLMTA facilitators using the WHO–AFRO–SLIPTA checklist. Two facilitators assessed six laboratories each, whilst one assessed seven. Training on the use of the checklist was conducted for two of the facilitators by the other facilitator who was a WHO trained assessor. The WHO–AFRO–SLIPTA checklist provides a quantitative measure of adherence to accreditation requirements for quality and competency. The scored checklist (totalling 250) allows for the rating of a laboratory’s quality improvement status by using a zero–five star rating, calculated as follows: 0–137 = zero stars, 138–160 = one star, 161–185 = two stars, 186–211 = three stars, 212–236 = four stars, and 237–250 = five stars.

### Improvement projects

For Cohort 2, a list of improvement projects was provided to participants at each workshop. These projects were based upon the findings of the baseline assessments that identified the weakest performing areas, as well as the follow-up visits. However, for Cohort 1, participants were allowed to select their own projects based on these baseline assessments. Table 3 lists the projects undertaken by the participants for Cohort 1 and Cohort 2.

**TABLE 3:** List of improvement projects carried out by Cohort 1 and Cohort 2 participants in the Strengthening Laboratory Management Towards Accreditation (SLMTA) programme in Lesotho.

Improvement project	Cohort 1	Cohort 2
1	Establish a document control system at QAU.	Reduce specimen rejection rate.
2	Improve the reporting of equipment breakdown and servicing to QAU by laboratories.	Improve turnaround time of test results.
3	Improve EQA participation of laboratories in Lesotho.	Improve IQC documentation (logs, reviews, corrective actions) in the CD4 testing section.
4	Improve performance scores on the general and safety audit by using the WHO–AFRO–SLIPTA checklist.	Improve EQA documentation (report reviews, investigation of poor performance and corrective actions).
5	Improve blood usage for transfusion.	Improve inventory management and decrease stock-outs.
6	Improve pap smear collection and transportation to Cytology.	Monitor and improve client satisfaction.
7	Improve result validation in Haematology at the Queen Elizabeth II Central Laboratory.	Implement visual cues in chemistry at Queen Elizabeth II Central Laboratory.
8	Establish IQC at Blood Transfusion Services.	Improve equipment maintenance performance and documentation at Motabang.
9	Improve waste management at blood transfusion services.	-
10	Determine CD4 sample stability for the Cyflow CD4 analyser at St Joseph’s.	-

QAU, Quality Assurance Unit; EQA, external quality assurance; IQC, internal quality control; WHO–AFRO–SLIPTA, World Health Organization Regional Headquarters for Africa Stepwise Laboratory Quality Improvement Toward Accreditation.

Participants were encouraged to share the tasks associated with the improvement project with the rest of the laboratory team. Each participant produced an improvement project report and prepared a PowerPoint presentation, whilst each participant’s laboratory was given a copy of the project write-up and another was retained by the QAU.

### Follow-up visits

For Cohort 2, each of the three facilitators was assigned laboratories which they followed throughout the duration of the programme. Each facilitator followed up with both the laboratories and the QE II Central Laboratory sections they had assessed at baseline. This allowed for relationship building between the facilitator and the laboratory. During each visit, the facilitator made an appointment with the hospital management to explain the vision of the Laboratory Directorate of Accreditation, as well as the SLMTA process. The need to support the laboratory was also emphasised.

A SLMTA follow-up visit assessment tool was developed and implemented for Cohort 2 to standardise follow-up visits and guide the facilitator on areas that needed to be covered during the site visit. These included the provision of supervision on the improvement project, follow-up on the implementation of activities taught during the last workshop by determining the uptake of SLMTA tools, the tracking of quality indicators and the implementation of a set of agreed compulsory activities. Compulsory activities were considered to be the ‘must do’ and easy-to-implement activities that did not constitute an improvement project, for example, a duty roster, an equipment master list, a team meeting and the use of a management calendar. In addition, the tool required the facilitator to document coaching provided to the SLMTA participant or other staff with regard to the improvement project, as well as in relation to other areas of laboratory improvement. Each visit lasted one full working day. All reports from visits were submitted to the SLMTA coordinator at the MOHSW, who ensured that the participating laboratories received copies of their progress reports.

## Results

A total of 12 participants were enrolled for Cohort 1 (Table 1), of whom 3 (25%) did not graduate. Of the nine who graduated, four were from each of the four district laboratories, four where affiliated with the QE II Central Laboratory sections of cytology, haematology, microbiology and blood transfusion, and one was from the QAU (Table 1). Of the three who did not graduate, one was transferred during the course of the training and could not continue, whilst the other two did not meet the requirements of 100% attendance and the completion of three improvement projects.

For Cohort 2, 19 participants were enrolled (Table 1), of whom 16 (84%) graduated. Three did not graduate because of reasons ranging from non-completion of three improvement projects and attending less than three workshops, to one participant being placed under disciplinary suspension.

**FIGURE 3:**
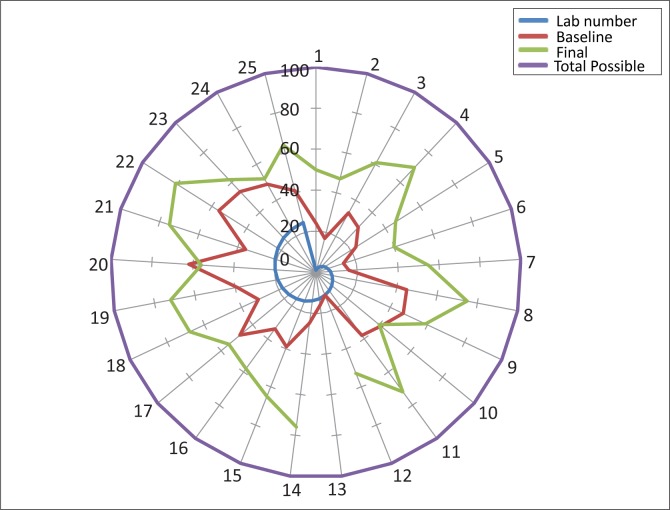
Performance of all laboratories at baseline and final assessments conducted in January 2010 and January 2011, respectively, using the World Health Organization Regional Headquarters for Africa Stepwise Laboratory Quality Improvement Toward Accreditation (WHO–AFRO–SLIPTA) checklist.

A total of 25 participants successfully completed the SLMTA programmes for Cohorts 1 and 2. These 25 were from 18 of the 19 laboratories of Lesotho. As mentioned above, the nineteenth laboratory, Quithin, missed the first workshop as a consequence of miscommunication of training dates. For assessment purposes, the QE II Central Laboratory had its seven sections of haematology, blood bank, cytology, blood transfusion, microbiology, TB laboratory and chemistry classified as separate sections; hence, the 25 assessments results in Figure 3.

### Improvement project outcomes

For Cohort 1, the participant from the QAU received the award for the best improvement projects. The selection was based on the overall impact that the participant’s three projects had on the entire laboratory service of Lesotho. The winner’s first project resulted in the upgrade and implementation of the current document control system for the entire network. Their second project improved the tracking of equipment down-time and the reporting of equipment breakdown for all contracted equipment within the government laboratory network, whilst their third project designed a mechanism of providing assessment for laboratories that miss the external quality assurance deadlines for various reasons.

**FIGURE 4:**
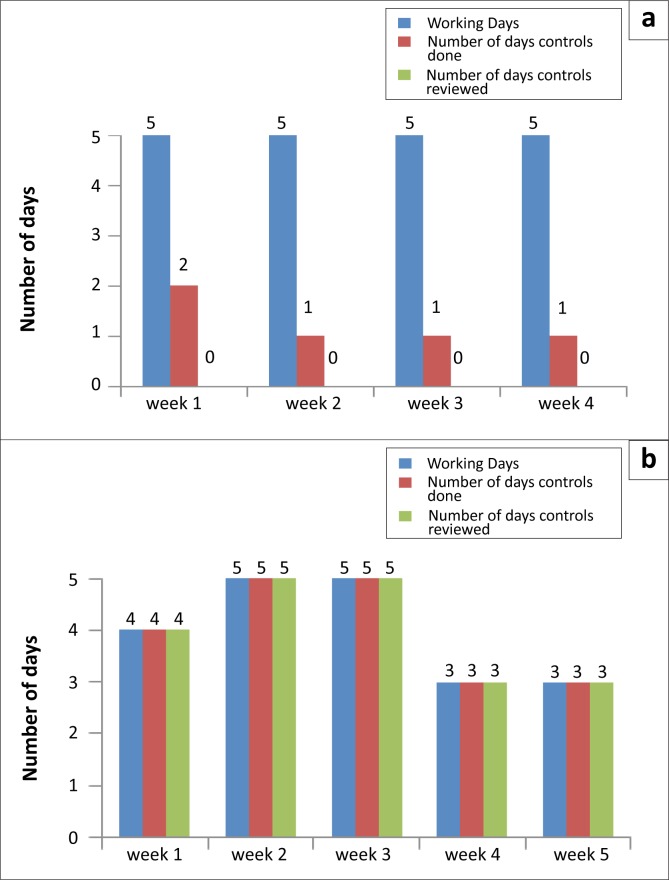
Example of an improvement project (from cohort 2) on improving performance and reviewing the internal quality control for CD4 the CD4 Section using a Cyflow analyser, indicating (a) the baseline data from February 2010 and (b) the final data at project completion in June 2010.

In Cohort 2, the Butha Buthe District Laboratory received the award for the best improvement project, which investigated the improvement of documentation in internal quality control for its CD4 section. The difference between baseline and final data collected between February and June 2010 for the Butha Buthe improvement project is illustrated in Figure 4.

The overall performance of laboratories over the entire SLMTA period improved over time (Figure 3). Of the 25 laboratories, 24 (96%) demonstrated an improvement over the 12-month period. One section at Queen II Central (Laboratory 13, Figure 3) was not assessed post-SLMTA because of miscommunication with the laboratory manager, which resulted in the assessor being unable to access the laboratory. The most improved laboratory demonstrated an increase of 51% from its baseline to final assessments, whilst the laboratory that performed the least demonstrated a 6% drop between these two assessments.

**FIGURE 5:**
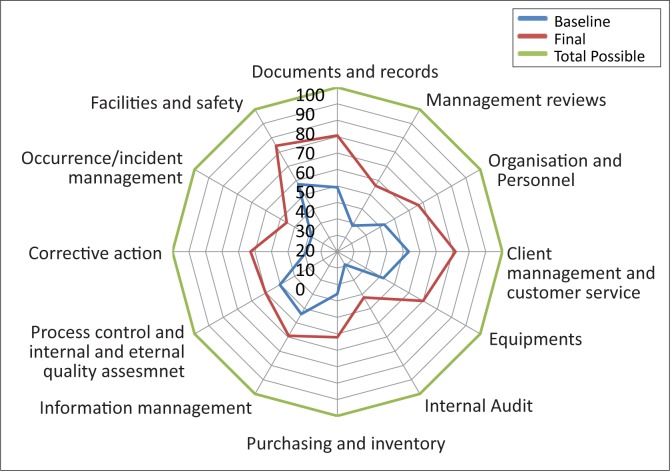
Average performance of all laboratories across the 12 sections, as measured by the World Health Organization Regional Headquarters for Africa Stepwise Laboratory Quality Improvement Toward Accreditation (WHO–AFRO–SLIPTA) checklist.

The average performance of the 25 laboratories across the 12 sections of the WHO–AFRO–SLIPTA checklist is illustrated in Figure 5. The most improved areas – measured by the difference between baseline and final percentage score for each of the 12 sections – were corrective actions (34%), documents and records (32%), and customer service (29%). The process improvement category demonstrated the least improvement (10%). The star rating of the laboratories using the WHO–AFRO–SLIPTA checklist star rating is reflected in Table 3. At baseline, all but one laboratory had zero stars; yet, by the end of SLMTA, 17 (68%) of the laboratories had achieved a star status. Of that 17, 8 (32%) had one star, 5 (20%) had two stars, and 4 (16%) had three stars, gained over a 12-month period of SLMTA (Table 4).

**TABLE 4:** Star rating of all laboratories between baseline and final assessments using the World Health Organization Regional Headquarters for Africa Stepwise Laboratory Quality Improvement Toward Accreditation (WHO–AFRO–SLIPTA) checklist.

Star rating	Baseline: January 2010	Final: January 2011^†^
0 stars	24	7
1 star	1	8
2 stars	0	5
3 stars	0	4
4 stars	0	0
5 stars	0	0

*Source*: Original data

† Tuberculosis laboratory was not assessed at the end of SLMTA.

## Discussion

Tracking of performance data by using the WHO–AFRO–SLIPTA checklist showed that only one (4%) of the 25 enrolled laboratories had at least a 1 star status rating at baseline out of a possible 5 stars. However, by January 2011, 17 (68%) had achieved a star rating, with four of the laboratories reaching three-star status. The results indicate that there was a measurable improvement over the 12-month period of SLMTA.

One laboratory (Laboratory 20, Figure 3) had a negative improvement of 6% because of an unexpected staff departure: the supervisor, who was in the SLMTA programme, was transferred to another duty station. Then, of the two technologists from Laboratory 20 who remained, one went on extended maternity leave. Even though systems could have been implemented, sustainment would have been difficult with only one out of three possible staff members in place. In addition, the Tebellong District Laboratory (Laboratory 10, Figure 3) had the smallest percentage of positive improvement, namely 1%, because of the unexpected withdrawal of an SLMTA participant. The participant had to be withdrawn from SLMTA because of his disciplinary suspension from the hospital and, as such, he could not attend Workshops 2 and 3. Tebellong had a staff complement of only two technologists. Replacement processes are carried out centrally at the MOHSW public services department and the process takes at least 6 months.

Laboratories, in general, were weaker in some areas than others. In particular, internal audits, management reviews, corrective actions and process improvement, showed the lowest average scores. These areas have been strengthened in SLMTA Cohort 3, currently in progress, as well as in the on-going mentorship programme.

One of the strongest pillars of success of SLMTA in Lesotho was the strong commitment shown by the Ministry of Health Laboratory Services to the SLMTA programme. The ownership and the strong leadership of the Directorate of Laboratory Services and its coordination of technical support by laboratory partners made the SLMTA successful in Lesotho. The high level of dedication demonstrated by SLMTA participants created tremendous enthusiasm within the laboratories, as observed by the three facilitators during the supervisory visits and workshop training. This also might have contributed to improvements, despite ever-increasing workloads.

Planning the entire SLMTA programme from the start helped to ensure that the programme was completed on time with few logistical problems. The entire 12-month programme for Cohort 2 was designed in January 2010, with fixed dates of baseline assessments, all three workshops, six follow-up visits and the final assessments decided upon at that time. This was critical, because SLMTA is a long and continuous process and therefore chances of disruptions are high. In this phase of SLMTA, 98% of the planned activities were met within the agreed timetable.

Coordination by the MOHSW was central to the success of SLMTA and functioned as a means of strengthening local capacity building within the QAU. Findings from the SLMTA assessments and site visits informed the QAU on priority areas. Standardising the supervisory visits and the meetings of hospital management for support was also critical for the improvement efforts. The MOHSW and its partners involved in the SLMTA as facilitators had to have dedicated time for the programme. The technical support and effective coordination of activities with the MOHSW by its partners (CHAI, APHL and CDC) also played a pivotal role in the rollout of the SLMTA programme.

## Conclusion

The SLMTA programme in Lesotho resulted in immediate, measurable laboratory improvements shown by all but one laboratory. With this performance, seven have been prepared and are ready for application to the WHO–AFRO–SLIPTA process.^4^ The seven selected are those with the highest WHO–AFRO–SLIPTA checklist marks from the SLMTA final assessments conducted by facilitators. As part of the preparation, these seven laboratories have already been assessed by WHO–AFRO–SLIPTA trained assessors who were invited to Lesotho in February 2011.

The Lesotho experience demonstrated that if SLMTA is planned and executed appropriately with the minimum of six follow-up visits and the three improvement projects, it will become an effective programme. Thus, it is clear that the improvement projects and follow-up visits are the two critical pillars of the SLMTA programme.

However, the programme did face some challenges. From the perception of the participants to the process, it was indicated that improvement projects consumed a lot of time and could not be carried out during the course of their normal working day. In some instances, participants had to request to be relieved from their routine work to work on the improvement projects. Only a few of the laboratories had computers for typing their projects and for drafting quality documents. Participants also felt that the time of 3 months allocated for improvement projects was too short. Furthermore, as a result of the strict criteria for SLMTA participation to graduation, there is a risk that participants may not be able to fulfil all criteria. This could result in the exclusion of laboratories from the SLMTA programme and a missed opportunity for laboratory improvement. This is another good reason to continue to roll out the SLMTA programme, as this will allow for continuous and sustainable laboratory quality improvement. This is the approach that Lesotho has taken by continuing the SLMTA training of more cohorts comprising different participants from the same laboratories.
